# miR-331-3p regulates expression of neuropilin-2 in glioblastoma

**DOI:** 10.1007/s11060-013-1271-7

**Published:** 2013-10-19

**Authors:** Michael R. Epis, Keith M. Giles, Patrick A. Candy, Rebecca J. Webster, Peter J. Leedman

**Affiliations:** 1 Laboratory for Cancer Medicine, Western Australian Institute for Medical Research and University of Western Australia Centre for Medical Research, Level 6, MRF Building, Rear 50 Murray Street, Perth, WA 6000 Australia; 2 School of Medicine and Pharmacology, University of Western Australia, Nedlands, WA 6008, Australia

**Keywords:** microRNA, Glioblastoma multiforme, miR-331-3p, Neuropilin-2

## Abstract

Aberrant expression of microRNAs (miRNAs), a class of small non-coding regulatory RNAs, has been implicated in the development and progression of high-grade gliomas. However, the precise mechanistic role of many miRNAs in this disease remains unclear. Here, we investigate the functional role of miR-331-3p in glioblastoma multiforme (GBM). We found that miR-331-3p expression in GBM cell lines is significantly lower than in normal brain, and that transient overexpression of miR-331-3p inhibits GBM cell line proliferation and clonogenic growth, suggesting a possible tumor suppressor role for miR-331-3p in this system. Bioinformatics analysis identified neuropilin-2 (NRP-2) as a putative target of miR-331-3p. Using transfection studies, we validated NRP-2 mRNA as a target of miR-331-3p in GBM cell lines, and show that NRP-2 expression is regulated by miR-331-3p. RNA interference (RNAi) to inhibit NRP-2 expression in vitro decreased the growth and clonogenic growth of GBM cell lines, providing further support for an oncogenic role for NRP-2 in high-grade gliomas. We also show that miR-331-3p inhibits GBM cell migration, an effect due in part to reduced NRP-2 expression. Finally, we identified a significant inverse correlation between miR-331-3p and NRP-2 expression in The Cancer Genome Atlas GBM cohort of 491 patients. Together, our results suggest that a loss of miR-331-3p expression contributes to GBM development and progression, at least in part via upregulating NRP-2 expression and increasing cell proliferation and clonogenic growth.

## Introduction

Glioblastoma multiforme (GBM) is the most common and lethal adult malignant brain tumor [1]. Despite advances in the standard therapy for GBM, which involves surgery followed by the combination of radiotherapy and temozolomide chemotherapy, tumor recurrence and treatment resistance are common and the median survival time of patients is only approximately 15 months [2]. Hence there is a pressing need to develop new approaches to treat this disease, and in recent years significant progress has been made in understanding the molecular biology of GBM, including the identification of aberrations in key cell signaling pathways that promote GBM tumorigenesis [2]. Furthermore, it has been shown that microRNAs (miRNAs)—short non-coding RNAs that repress gene expression by binding to specific target mRNAs—have altered expression in a variety of cancers, including GBM (reviewed in [3]), where they may act as oncogenes or tumor suppressor genes. For example, miR-21 is overexpressed in GBM and promotes tumor cell growth, survival and invasion [4–6], and inhibition of miR-21 sensitizes GBM cells to temozolomide chemotherapy [7]. Reduced expression of tumor suppressor miRNAs, including miR-7, miR-34a, and the let-7 miRNA family promotes oncogenic signaling in GBM [8–10]. These findings and others suggest not only the potential for miRNAs to serve as prognostic and diagnostic molecules in GBM, but also that the delivery of inhibitors of oncogenic miRNAs and/or replacement therapy using mimics of tumor suppressor miRNAs represents a novel strategy to treat GBM (reviewed in [11]).

Previously, we and others identified miR-331-3p as a tumor suppressor miRNA that is downregulated in prostate cancer, where it mediates ErbB-2 expression and PI3K/Akt signaling [12, 13], as well as expression of deoxyhypusine hydroxylase (DOHH), an enzyme that activates eukaryotic translation initiation factor (eIF5A) and regulates cell growth [14]. Following a report that miR-331-3p expression was reduced in a panel of central nervous system tumor cell lines [6], we investigated the expression and function of miR-331-3p in GBM. In this paper, we show that miR-331-3p expression is decreased in a panel of GBM cell lines relative to normal brain tissue and that miR-331-3p inhibits the growth and clonogenicity of GBM cell lines in vitro. Furthermore, we show that miR-331-3p inhibits expression of neuropilin-2 (NRP-2), a receptor implicated in neuronal development, axon guidance and tumorigenesis, and that NRP-2 promotes the growth and clonogenicity of GBM cell lines in vitro. Transfection with miR-331-3p also suppresses GBM cell migration in vitro, in part by its inhibition of NRP-2 expression. Finally, analysis of miRNA and mRNA expression data from The Cancer Genome Atlas (TCGA) GBM cohort shows an inverse correlation between miR-331-3p and NRP-2 levels in clinical GBM samples. Our findings suggest that miR-331-3p may have prognostic or therapeutic utility in the management of GBM.

## Experimental procedures

### Cell culture, plasmid DNA, miRNA precursor and siRNA molecules

U-251 MG, U-373 MG and U-87 MG glioblastoma cell lines were obtained from the American Type Culture Collection (ATCC) and cultured at 37 °C in 5 % CO_2_ with RPMI-1640 supplemented with 10 % fetal bovine serum. The NRP-2 3′-UTR and negative control reporter clones were obtained from Gencopoeia (NRP-2 reporter; Cat# HmiT021592-MT01: Negative control reporter; Cat # CmiT000001-MT01). All plasmids were verified by DNA sequencing prior to use. Synthetic miRNA precursor molecules corresponding to human miR-331-3p (pre-miR miRNA Precursor Product ID: PM10881 and negative control miRNA (miR-NC; pre-miR miRNA precursor negative control #1, Product ID: AM17110) were obtained from Ambion. Synthetic siRNA precursor molecules corresponding to human NRP-2 (Silencer Select si-NRP-2; Cat #4390824 ID:16840) and siRNA negative control molecules (Silencer Select si-NC; Cat #4390843) were obtained from Ambion.

### RNA extraction, reverse transcription and quantitative polymerase chain reaction (RT-qPCR)

Total normal brain RNA was obtained from Ambion (Cat #AM7962) and used as a reference control sample. Total RNA from GBM cell lines was extracted using Qiazol reagent (Qiagen). For miRNA detection, quantitative Taqman qRT-PCR was performed using TaqMan miRNA assays (Life Technologies) for hsa-miR-331-3p and RNU6B small nuclear RNA (Life Technologies; Assay IDs 000545 and 001093), with 10 ng total RNA. Reactions were carried using a Rotor-Gene 6000 thermocycler (Qiagen). Expression of mature miR-331-3p relative to RNU6B small nuclear RNA (snRNA) was determined using the 2^−ΔΔCt^ method [15]. Statistical analysis of RT-qPCR data was performed using GenEx software (MultiD).

### Transfection of siRNA/miRNA precursor molecules and reporter gene assays

GBM cells were seeded into 12 well or 10 cm dishes and transfected with siRNA or miRNA precursor molecules at a final concentration of 5 nM and 1–30 nM, respectively. Briefly, GBM cells were transfected with siRNA to NRP-2 (NRP-2 Silencer Select siRNA ID:S16840) or Negative Control (Negative Control #1 Silencer Select siRNA) at a final concentration of 5 nM, also using Lipofectamine 2000 (Invitrogen). Cells were harvested after 48 h for protein extraction.

Reporter gene assays were performed as described [16]. GBM cells were seeded in 12-well plates and transfected with 100 ng of firefly luciferase reporter plasmid DNA (NRP-2 or Negative control; Gencopoeia) and miRNA precursor (Ambion; pre-miR-331-3p or pre-miR-NC; 1–5 nM final concentration), using Lipofectamine 2000. After 24 h, lysates were assayed for firefly and *Renilla* luciferase activities using the dual luciferase reporter assay system (Promega) and a Fluostar OPTIMA microplate reader (BMG Labtech). Firefly luciferase activity for each sample was normalized to *Renilla* luciferase activity to yield a relative luciferase activity.

### Protein extraction and western blotting

Cytoplasmic protein extracts were prepared and western blotting performed as described [16]. Briefly, protein samples were resolved on NuPAGE 4–12 % Bis Tris gels (Invitrogen) and transferred to PVDF membranes (Roche). Membranes were blocked in 5 % skim milk/TBST and probed with either anti-tubulin rat polyclonal antibody (1:1,000, Abcam ab6161-100), anti-β-actin mouse monoclonal antibody (1:10,000, Abcam ab6276-100), anti NRP-2 (C-9) mouse polyclonal antibody (Santa Cruz Biotechnology sc-13117) or anti-DOHH (C-19) goat polyclonal antibody (1:1,000, Santa Cruz Biotechnology sc-55157). Detection was performed with horseradish peroxidise-linked anti-rat-IgG (1:10,000; ab6734-1; Abcam), anti-mouse-IgG (1:10,000; NA931 V; GE Healthcare) and anti-sheep/goat-IgG (1:10,000; AB324P; Chemicon) secondary antibodies with ECL Plus detection reagent and ECL-Hyperfilm (GE Healthcare).

### Clonogenicity, cell proliferation and migration assays

Clonogenicity assays were performed using U-251 MG and U-373 MG cells transiently transfected with miR-NC or miR-331-3p as described previously [17]. For cell titre assays, GBM cells were transfected with miRNA precursor molecules (see above) and cell proliferation was assessed at 7 days post transfection with the CellTiter 96 Aqueous One Solution Cell Proliferation System (Promega) and a Fluostar Optima plate reader (BMG Scientific). The xCELLigence real time proliferation system (Roche) was used to detect changes in proliferation of GBM cells over a 120 h period in real time. Briefly, GBM cells were transfected with miRNA precursor molecules and trypsinized and counted after 48 h. E-plate16 xCELLigence plates were prepared as per manufacturer’s recommendations and transfected GBM cells added to wells in groups of 4 wells per treatment. Cell proliferation was measured by a calculated cell index using the xCELLigence device. At the completion of the experiment, cell proliferation curves were normalised at 10 h post seeding (where true t = 0 was at t = 10 h; see Fig. 3b) of cells in the E-plate16 to the miR-NC transfected cells, to achieve the same base line cell index for both miR-NC and miR-331-3p transfected GBM cells in the assay. Migration of transfected U-251 MG cells in the CIM-plate16 was monitored with an xCELLigence real time migration system (Roche) as described previously ([18]).

### Statistical and scatterplot analyses

Statistical analysis of RT-qPCR data was performed using GENEX software (MultiD). All analyses were performed at a minimum confidence interval of 95 % (CI 0.95) and normality of data confirmed by Kolmogorov-Smirnoff test (KS Test). Statistical analysis of reporter gene assay data was performed using Student’s *t* test, where *p* < 0.05 represented a significant difference. Error bars represent standard deviations (SD).

To investigate the relationship between the expression of miR-331-3p and NRP-2 in human GBM and normal brain tissue, we utilized data from TCGA [19]. We queried the TCGA data portal (https://tcga-data.nci.nih.gov/tcga/, accessed March 2013) for all GBM and normal brain tissue samples with Level 3 miRNA (Agilent 8 × 15 K Human miRNA-specific Microarray) and gene (Affymetrix HT Human Genome U133 Array Plate Set) expression data available. The resulting data set was filtered for samples having expression data for both miR-331-3p and NRP-2, yielding a final set of 491 independent patient samples, including 482 GBM and 9 unmatched normal brain samples. Statistical analyses were performed using the statistical analysis package, R [20]. A simple linear model was fit to the miR-331-3p and NRP-2 expression data using the *lm* function.

## Results

### miR-331-3p expression in GBM cell lines is significantly lower than in normal brain and miR-331-3p inhibits proliferation and clonogenic growth of GBM cell lines

**Fig. 1:**
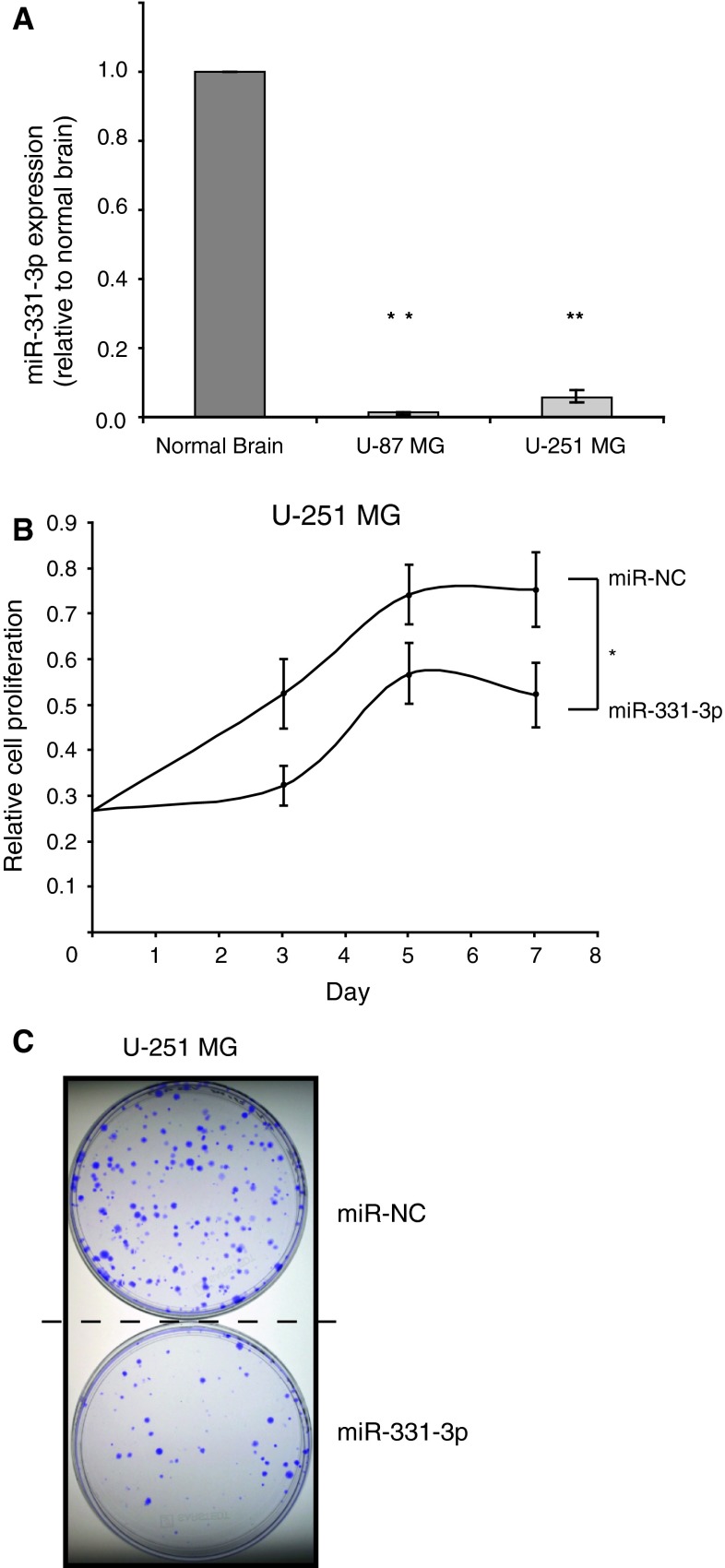
miR-331-3p is down regulated in GBM cell lines compared to normal brain and inhibits cell proliferation and clonogenicity. **a** TaqMan RT-qPCR analysis of miR-331-3p expression in U-87 MG and U-251 MG cells relative to normal brain RNA. **b** Cell titre assay of U-251 MG cell proliferation following transient transfection with either miR-331-3p or negative control miRNA (miR-NC). **c** Clonogenicity assay of U-251 MG cells 12 days following transient transfection with either miR-331-3p or miR-NC. *Error bars* represent standard deviations; *p < 0.05, **p < 0.01

Previously, we demonstrated a tumor suppressor role for miR-331-3p in prostate cancer, where miR-331-3p expression is reduced in a cohort of patient tumors relative to matched normal adjacent tissue [12, 14]. Following a report by Gaur and co-workers [6] which demonstrated that miR-331-3p expression is decreased in a panel of CNS tumor cell lines (SNB19, SF295, SF539, U-251 MG, SF268, SNB75) relative to normal brain tissue, we used TaqMan miRNA RT-qPCR assays to determine miR-331-3p levels in U-87 MG and U-251 MG GBM cell lines compared with normal brain RNA. Our data are consistent with the above-mentioned report and show that miR-331-3p expression is significantly lower in both GBM cell lines relative to normal brain (Fig. 1a), suggesting that it may have a tumor suppressor role in this system.

To investigate the functional significance of miR-331-3p loss in GBM, we transiently transfected U-251 MG and U-373 MG cells with either a synthetic miR-331-3p mimic or a negative control miRNA mimic (miR-NC), and measured cell viability over time as a measure of cell proliferation. Our data indicate that miR-331-3p transfection reduced cell proliferation over a 7 day period (Fig. 1b for U-251 MG, and data not shown for U-373 MG cells). To support this finding, we assessed the clonogenic growth of U-251 MG cells transfected with either miR-331-3p or miR-NC, and found that transfection of miR-331-3p suppressed colony formation over a 3 week period (Fig. 1c). Taken together, our results suggest that down regulation of miR-331-3p may promote the growth of GBM cells.

### Neuropilin-2 (NRP-2) is targeted by miR-331-3p in GBM cell lines

To assess the tumor suppressive action of miR-331-3p in GBM cells, we used miRNA target prediction software (TargetScan Release 6.2, June 2012) [21] to identify putative miR-331-3p target mRNAs that could mediate its anti-proliferative effects in GBM. Using a similar approach we previously identified ErbB-2 and Deoxyhypusine hydroxylase (DOHH) as novel targets of miR-331-3p in prostate cancer [12, 14]. TargetScan analysis indicated that the second highest confidence predicted target of miR-331-3p is NRP-2.

**Fig. 2:**
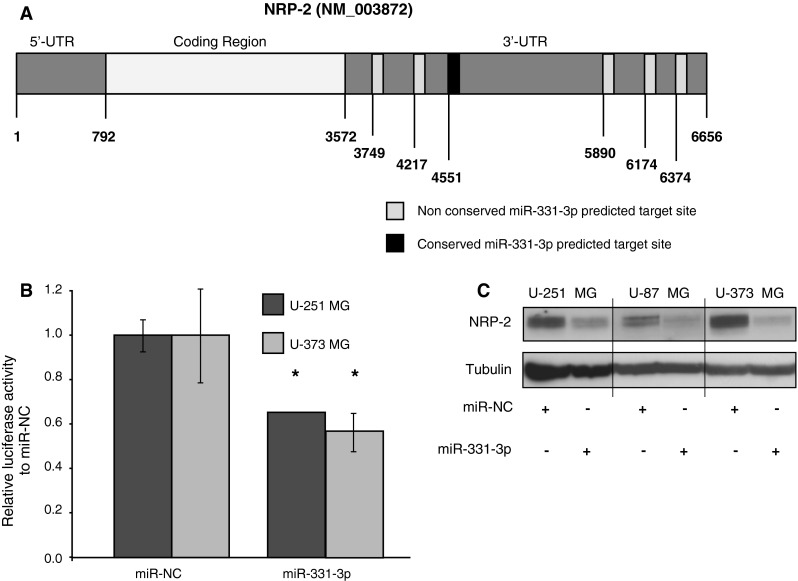
NRP-2 expression is regulated by miR-331-3p in GBM cells. **a** Schematic representation of the NRP-2 mRNA, showing the 3′-UTR containing six predicted miR-331-3p binding sites (TargetScan version 6.2, June 2012). Nucleotide positions of miR-331-3p seed sequence sites are shown within the 3′-UTR, with boxes shaded light for non-conserved sites and dark for conserved sites. **b** Luciferase reporter assay using U-251 MG and U-373 MG cells co-transfected with full length NRP-2 3′-UTR luciferase reporter gene plasmid DNA and either miR-331-3p or miR-NC. For each sample, firefly luciferase activity was normalized to *Renilla* luciferase activity, and data is expressed relative to each cell line cotransfected with reporter plasmid DNA and miR-NC. Data for each reporter construct is expressed relative to miR-NC transfected cells. *Error bars* represent standard deviations; *p < 0.05. **c** Western blotting analysis of NRP-2 protein expression in U-251 MG, U-87 MG and U-373 MG GBM cells 48 h after miR-331-3p or miR-NC transfection. α-tubulin is included as a loading control

Analysis of the 3′-untranslated region (3′-UTR) of NRP-2 revealed six putative miR-331-3p target sites (Fig. 2a and Table 1), one of which is conserved across species. To determine whether miR-331-3p could target the NRP-2 3′-UTR, we co-transfected U-251 MG and U-373 MG cells with a luciferase reporter construct containing the full length NRP-2 3′-UTR and either miR-331-3p or miR-NC. We observed that miR-331-3p significantly decreased reporter gene activity relative to miR-NC (Fig. 2b), indicating that the NRP-2 3′-UTR contains functional target sites for binding and activity of miR-331-3p. Furthermore, transfection of U-251 MG, U-87 MG and U-373 MG cells with miR-331-3p inhibited expression of endogenous NRP-2 protein by western blotting (Fig. 2c). As a positive control for this assay we assessed the expression of DOHH, a validated miR-331-3p target in prostate cancer cells [14] and found that DOHH expression was decreased in GBM cells following transfection of miR-331-3p (data not shown). Together, our results indicate that miR-331-3p is a potent regulator of NRP-2 expression in GBM cells.

**Table 1:** miR-331-3p seed regions and their respective TargetScan context scores within the NRP-2 3′-UTR

miR-331-3p seed region in NRP-2 3′-UTR NRP-2	Context score (%)	Conserved or poorly conserved
Position 177–183	46	Poorly conserved
Position 645–652	97	Conserved
Position 979–985	29	Poorly conserved
Position 2,318–2,324	57	Poorly conserved
Position 2,602–2,609	91	Poorly conserved
Position 2802–2808	43	Poorly conserved

### NRP-2 regulates proliferation and clonogenic growth of GBM cell lines

**Fig. 3:**
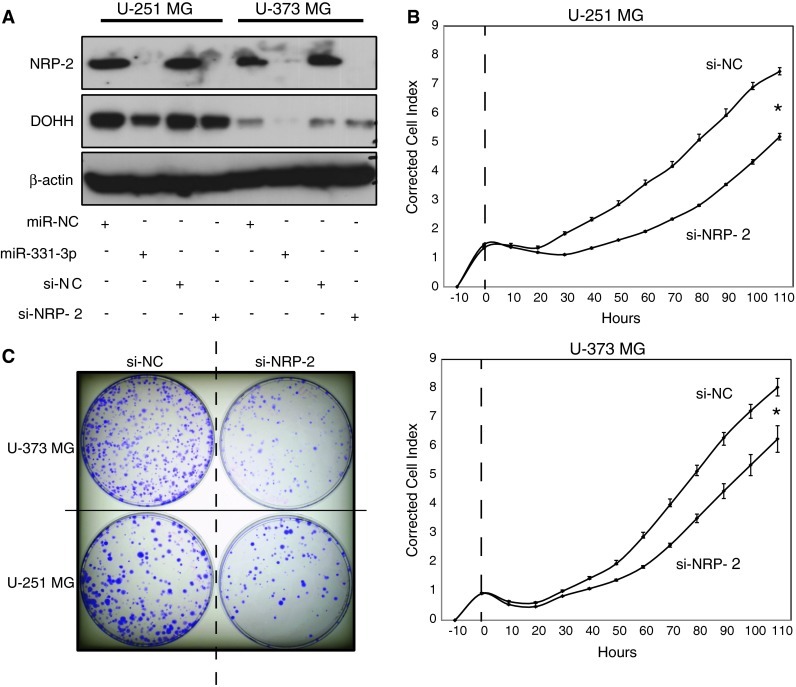
NRP-2 promotes GBM cell proliferation and clonogenicity. **a** Western blotting analysis of NRP-2 and DOHH protein expression in U-251 MG and U-373 MG GBM cells 48 h after transfection with miR-331-3p, miR-NC, si-NRP-2, or si-NC (negative control siRNA). DOHH was included as a positive control (known miR-331-3p target), and β-actin was included as a loading control. **b** xCELLigence assay of U-251 MG and U-373 MG cell proliferation in real time following transient transfection with either si-NRP-2 or si-NC. Data was normalized to measured cell index at 10 h post-seeding of cells in xCELLigence plates. **c** Clonogenicity assay of U-373 MG and U-251 MG cells following transient transfection with either si-NRP-2 or si-NC. Colonies were fixed with ice-cold methanol and stained with crystal violet 12 days after plating of cells. *Error bars* represent standard deviations; *p < 0.05

To validate NRP-2 as a functional target of miR-331-3p in GBM, we used RNAi to inhibit NRP-2 expression in U-251 MG and U-373 MG cells. We first confirmed that NRP-2 RNAi decreased NRP-2 expression by western blotting relative to negative control siRNA (Fig. 3a). As a control for this experiment, we also transfected U-251 MG and U-373 MG cells with either miR-331-3p or miR-NC and observed reduced NRP-2 expression with miR-331-3p (Fig. 3a). We next assessed the impact of NRP-2 RNAi on the proliferation and clonogenic growth of U-251 MG and U-373 MG cells. Using a real-time xCELLigence assay, transfection of each cell line with NRP-2 siRNA (si-NRP-2) significantly inhibited proliferation over a 120 h period relative to non-targeting siRNA (si-NC) (Fig. 3b). In addition, NRP-2 siRNA transfectants exhibited reduced colony formation over a 14 day period (Fig. 3c). The similarity in effects on proliferation and clonogenicity between NRP-2 RNAi and miR-331-3p transfection (Fig. 1) suggest that NRP-2 may be a key functional target of miR-331-3p in GBM.

### Inhibition of NRP-2 by miR-331-3p or NRP-2 siRNA represses the migration of U-251 MG GBM cells

**Fig. 4:**
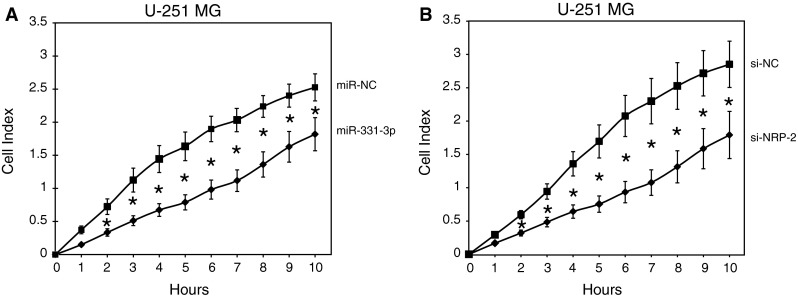
Reduced migration of U-251 MG cells following transfection with miR-331-3p or siRNA to NRP-2. xCELLigence assay of U-251 MG cell migration in real time following transient transfection with either **a** miR-331-3p or **b** si-NRP-2. Data is expressed relative to miR-NC or si-NC. *Error bars* represent standard deviations; *p < 0.05

As NRP-2 is known to promote GBM cell motility [22], we tested the capacity for miR-331-3p to regulate migration of U-251 MG cells in vitro using xCELLIGENCE real-time migration assays. Transfection with either miR-331-3p or NRP-2 siRNA significantly repressed migration of U-251 MG cells (Fig. 4a, b), and suggested that the anti-migratory effect of miR-331-3p is at least in part mediated by its repression of NRP-2 expression.

### NRP-2 expression is inversely correlated with miR-331-3p expression in a GBM patient cohort

**Fig. 5:**
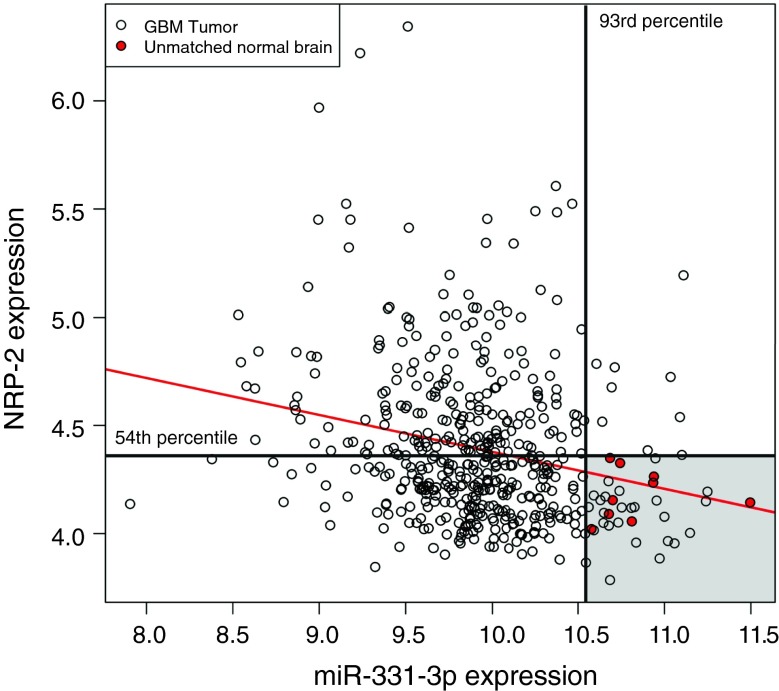
Inverse association between expression of miR-331-3p and NRP-2 in The Cancer Genome Atlas (TCGA) GBM cohort. Scatterplot of miR-331-3p and NRP-2 expression in GBM (*unfilled circles*) and unmatched normal brain (*red-filled circles*) tissue samples from TCGA with fitted regression line (*red line*). The 93rd percentile of miR-331-3p expression in the GBM samples (*vertical black line*) and 54th percentile of NRP-2 expression in the GBM samples (*horizontal black line*) delineate the range of expression values of the normal brain samples (*shaded grey area*). *Scale* indicates NRP-2 (*vertical*) and miR-331-3p (*horizontal*) probe intensities

The expression of miR-331-3p and NRP-2 in GBM and normal brain tissue samples from TCGA was visualized with a scatterplot (Fig. 5). A linear model fitted to this data indicates a significant inverse relationship between miR-331-3p and NRP-2 expression (*p* = 1.5 × 10^−7^), with a correlation coefficient of r = −0.23 (r^2^ = 0.055). All normal brain samples had miR-331-3p expression values above the 93rd percentile of the GBM samples, with only 34 of 482 GBM samples (7 %) having values in the same range. NRP-2 expression values in normal brain samples were all below the 54th percentile of the GBM samples with 260 of 482 GBM samples (54 %) having values in this range. Only 24 of 482 GBM samples (5 %) had both miR-331-3p and NRP-2 expression in the same range as the normal brain samples.

## Discussion

In this study we have investigated the significance of miR-331-3p regulation of NRP-2 in GBM. We found several lines of evidence to suggest that miR-331-3p is a tumor suppressor miRNA in GBM. Firstly, we identified low miR-331-3p expression in GBM cell lines compared to normal brain. Secondly, transfection of miR-331-3p decreased proliferation and clonogenic growth of GBM cell lines. Our results provide evidence that NRP-2 is a target of miR-331-3p, and we show that inhibition of NRP-2 by RNAi attenuates GBM cell proliferation and clonogenicity. Furthermore, miR-331-3p inhibits GBM cell migration, an effect that can in part be attributed to its suppression of NRP-2 expression. Finally, analysis of 491 GBM and normal brain tissue samples from TCGA supported an inverse relationship between miR-331-3p and NRP-2 expression, consistent with NRP-2 being a target of miR-331-3p.

A number of tumor suppressor miRNAs have been identified as being downregulated in GBM; including miR-7, miR-34a, miR-128, and miR-137 (reviewed in [23]). In addition to demonstrating reduced expression of miR-331-3p in GBM cell lines and patient tumors, we previously found that miR-331-3p expression is reduced in prostate cancer tissue relative to matched normal adjacent tissue [12, 14]. The human *miR*-*331* gene is located at 12q22, a chromosomal region that is frequently lost in GBM [24]. It would be interesting to correlate expression of the miR-331-3p primary transcript with 12q22 loss in GBM. We and others identified several targets of miR-331-3p in prostate cancer cells, including ErbB-2 [12], DOHH [14] and KLK4 [25]. It was also proposed that miR-331-3p may coordinately regulate cell cycle progression in gastric cancer cells [26]. Interestingly, in the present study we observed that expression of DOHH, an enzyme involved in the activation of eIF5A and control of cell cycle progression [27], is regulated by miR-331-3p in GBM cell lines. A recent report by Preukschas and coworkers showed that DOHH is highly overexpressed in GBM patient tissues, and that inhibition of DOHH action in GBM cell lines caused cell cycle arrest and sensitized cells to clinically relevant alkylating agents [28]. Taken together, these findings suggest that miR-331-3p acts as a tumor suppressor in the brain by coordinately inhibiting expression of multiple target molecules, including NRP-2 and DOHH, leading to reduced GBM cell proliferation and clonogenic growth.

The capacity for miR-331-3p to regulate NRP-2 expression is of interest given a number of recent reports that emphasize the importance of NRP-2 in tumor growth and progression, and as a promising therapeutic target. CNS tumors (e.g. neuroblastoma) frequently express NRP-2 [29], and NRP-2 expression is also elevated in high-grade prostate cancers with PTEN loss [30], a frequent event in glioblastoma [31]. NRP-2 is essential for breast cancer tumor initiation [32], where it is involved in the formation of focal adhesions [33] and is associated with metastasis and poor prognosis [34]. NRP-2 also promotes the invasion and migration of thyroid cancer cells in vitro, an effect that could be blocked by a neutralizing anti-NRP-2 antibody [35]. Furthermore, combined blockade of NRP-2 action and treatment with bevacizumab anti-angiogenic therapy produced synergistic inhibition of melanoma xenograft growth [36], a finding that has implications for GBM treatment given the emerging therapeutic role of bevacizumab in this disease [37]. Treatment of mice with NRP-2 siRNA inhibited the growth of colorectal cancer hepatic metastases, providing proof-of-principle for treatment with small RNA molecules against NRP-2 [38]. It would be of interest to evaluate the anti-tumor efficacy of systemic or local miR-331-3p delivery, particularly given the apparent feasibility of miRNA-based therapies in various CNS tumor types [3].

In summary, we have identified miR-331-3p as a novel regulator of NRP-2 in GBM. Elevated NRP-2 expression is correlated with low levels of miR-331-3p in clinical GBM samples. miR-331-3p acts at least in part via blocking NRP-2 to inhibit GBM cell proliferation, clonogenic growth, and migration. These results warrant further investigation into both the functional roles and the therapeutic and prognostic utility of these molecules in GBM.

## References

[1] Bai RY, Staedtke V, Riggins GJ (2011). Molecular targeting of glioblastoma: drug discovery and therapies. Trends Mol Med.

[2] Mao H, Lebrun DG, Yang J, Zhu VF, Li M (2012). Deregulated signaling pathways in glioblastoma multiforme: molecular mechanisms and therapeutic targets. Cancer Invest.

[3] Nikaki A, Piperi C, Papavassiliou AG (2012). Role of microRNAs in gliomagenesis: targeting miRNAs in glioblastoma multiforme therapy. Expert Opin Investig Drugs.

[4] Chan JA, Krichevsky AM, Kosik KS (2005). MicroRNA-21 is an antiapoptotic factor in human glioblastoma cells. Cancer Res.

[5] Gabriely G, Wurdinger T, Kesari S, Esau CC, Burchard J, Linsley PS, Krichevsky AM (2008). microRNA 21 promotes glioma invasion by targeting matrix metalloproteinase regulators. Mol Cell Biol.

[6] Gaur A, Jewell DA, Liang Y, Ridzon D, Moore JH, Chen CF, Ambros VR, Israel MA (2007). Characterization of microRNA expression levels and their biological correlates in human cancer cell lines. Cancer Res.

[7] Wong ST, Zhang XQ, Zhuang JT, Chan HL, Li CH, Leung GK (2012). microRNA-21 inhibition enhances in vitro chemosensitivity of temozolomide-resistant glioblastoma cells. Anticancer Res.

[8] Lee ST, Chu K, Oh HJ, Im WS, Lim JY, Kim SK, Park CK, Jung KH, Lee SK, Kim M, Roh JK (2012). Let-7 microRNA inhibits the proliferation of human glioblastoma cells. J Neurooncol.

[9] Li Y, Guessous F, Zhang Y, Dipierro C, Kefas B, Johnson E, Marcinkiewicz L, Jiang J, Yang Y, Schmittgen TD, Lopes B, Schiff D, Purow B, Abounader R (2009). microRNA-34a inhibits glioblastoma growth by targeting multiple oncogenes. Cancer Res.

[10] Webster RJ, Giles KM, Price KJ, Zhang PM, Mattick JS, Leedman PJ (2009). Regulation of epidermal growth factor receptor signaling in human cancer cells by microRNA-7. J Biol Chem.

[11] Purow B (2011). The elephant in the room: do microRNA-based therapies have a realistic chance of succeeding for brain tumors such as glioblastoma?. J Neurooncol.

[12] Epis MR, Giles KM, Barker A, Kendrick TS, Leedman PJ (2009). miR-331-3p regulates ERBB-2 expression and androgen receptor signaling in prostate cancer. J Biol Chem.

[13] Wang L, Tang H, Thayanithy V, Subramanian S, Oberg AL, Cunningham JM, Cerhan JR, Steer CJ, Thibodeau SN (2009). Gene networks and microRNAs implicated in aggressive prostate cancer. Cancer Res.

[14] Epis MR, Giles KM, Kalinowski FC, Barker A, Cohen RJ, Leedman PJ (2012). Regulation of expression of deoxyhypusine hydroxylase (DOHH), the enzyme that catalyzes the activation of eIF5A, by miR-331-3p and miR-642-5p in prostate cancer cells. J Biol Chem.

[15] Livak KJ, Schmittgen TD (2001). Analysis of relative gene expression data using real-time quantitative PCR and the 2(-Delta Delta C(T)) Method. Methods.

[16] Giles KM, Barker A, Zhang PM, Epis MR, Leedman PJ (2011). microRNA regulation of growth factor receptor signaling in human cancer cells. Methods Mol Biol.

[17] Kalinowski FC, Giles KM, Candy PA, Ali A, Ganda C, Epis MR, Webster RJ, Leedman PJ (2012). Regulation of epidermal growth factor receptor signaling and erlotinib sensitivity in head and neck cancer cells by miR-7. PLoS ONE.

[18] Giles KM, Brown RA, Epis MR, Kalinowski FC, Leedman PJ (2013). miRNA-7-5p inhibits melanoma cell migration and invasion. Biochem Biophys Res Commun.

[19] Cancer Genome Atlas Research N (2008). Comprehensive genomic characterization defines human glioblastoma genes and core pathways. Nature.

[20] Ihaka R, Gentleman R (1996). R: a language for data analysis and graphics. J Comput Graph Stat.

[21] Lewis BP, Burge CB, Bartel DP (2005). Conserved seed pairing, often flanked by adenosines, indicates that thousands of human genes are microRNA targets. Cell.

[22] Mariani L, Beaudry C, McDonough WS, Hoelzinger DB, Demuth T, Ross KR, Berens T, Coons SW, Watts G, Trent JM, Wei JS, Giese A, Berens ME (2001). Glioma cell motility is associated with reduced transcription of proapoptotic and proliferation genes: a cDNA microarray analysis. J Neurooncol.

[23] Moller HG, Rasmussen AP, Andersen HH, Johnsen KB, Henriksen M, Duroux M (2013). A systematic review of microRNA in glioblastoma multiforme: micro-modulators in the mesenchymal mode of migration and invasion. Mol Neurobiol.

[24] Watanabe T, Hirota Y, Arakawa Y, Fujisawa H, Tachibana O, Hasegawa M, Yamashita J, Hayashi Y (2003). Frequent LOH at chromosome 12q22-23 and Apaf-1 inactivation in glioblastoma. Brain Pathol.

[25] White NM, Youssef YM, Fendler A, Stephan C, Jung K, Yousef GM (2012). The miRNA-kallikrein axis of interaction: a new dimension in the pathogenesis of prostate cancer. Biol Chem.

[26] Guo X, Guo L, Ji J, Zhang J, Zhang J, Chen X, Cai Q, Li J, Gu Q, Liu B, Zhu Z, Yu Y (2010). miRNA-331-3p directly targets E2F1 and induces growth arrest in human gastric cancer. Biochem Biophys Res Commun.

[27] Park JH, Aravind L, Wolff EC, Kaevel J, Kim YS, Park MH (2006). Molecular cloning, expression, and structural prediction of deoxyhypusine hydroxylase: a HEAT-repeat-containing metalloenzyme. Proc Natl Acad Sci USA.

[28] Preukschas M, Hagel C, Schulte A, Weber K, Lamszus K, Sievert H, Pallmann N, Bokemeyer C, Hauber J, Braig M, Balabanov S (2012). Expression of eukaryotic initiation factor 5A and hypusine forming enzymes in glioblastoma patient samples: implications for new targeted therapies. PLoS ONE.

[29] Fakhari M, Pullirsch D, Abraham D, Paya K, Hofbauer R, Holzfeind P, Hofmann M, Aharinejad S (2002). Selective upregulation of vascular endothelial growth factor receptors neuropilin-1 and -2 in human neuroblastoma. Cancer.

[30] Goel HL, Chang C, Pursell B, Leav I, Lyle S, Xi HS, Hsieh CC, Adisetiyo H, Roy-Burman P, Coleman IM, Nelson PS, Vessella RL, Davis RJ, Plymate SR, Mercurio AM (2012). VEGF/neuropilin-2 regulation of Bmi-1 and consequent repression of IGF-IR define a novel mechanism of aggressive prostate cancer. Cancer Discov.

[31] Tohma Y, Gratas C, Biernat W, Peraud A, Fukuda M, Yonekawa Y, Kleihues P, Ohgaki H (1998). PTEN (MMAC1) mutations are frequent in primary glioblastomas (de novo) but not in secondary glioblastomas. J Neuropathol Exp Neurol.

[32] Goel HL, Pursell B, Chang C, Shaw LM, Mao J, Simin K, Kumar P, Vander Kooi CW, Shultz LD, Greiner DL, Norum JH, Toftgard R, Kuperwasser C, Mercurio AM (2013). GLI1 regulates a novel neuropilin-2/alpha6beta1 integrin based autocrine pathway that contributes to breast cancer initiation. EMBO Mol Med.

[33] Goel HL, Pursell B, Standley C, Fogarty K, Mercurio AM (2012). Neuropilin-2 regulates alpha6beta1 integrin in the formation of focal adhesions and signaling. J Cell Sci.

[34] Yasuoka H, Kodama R, Tsujimoto M, Yoshidome K, Akamatsu H, Nakahara M, Inagaki M, Sanke T, Nakamura Y (2009). Neuropilin-2 expression in breast cancer: correlation with lymph node metastasis, poor prognosis, and regulation of CXCR4 expression. BMC cancer.

[35] Yasuoka H, Kodama R, Hirokawa M, Takamura Y, Miyauchi A, Inagaki M, Sanke T, Nakamura Y (2011). Neuropilin-2 expression in papillary thyroid carcinoma: correlation with VEGF-D expression, lymph node metastasis, and VEGF-D-induced aggressive cancer cell phenotype. J Clin Endocrinol Metab.

[36] Geretti E, van Meeteren LA, Shimizu A, Dudley AC, Claesson-Welsh L, Klagsbrun M (2010). A mutated soluble neuropilin-2 B domain antagonizes vascular endothelial growth factor bioactivity and inhibits tumor progression. Mol Cancer Res.

[37] Specenier P (2012). Bevacizumab in glioblastoma multiforme. Expert Rev Anticancer Ther.

[38] Gray MJ, Van Buren G, Dallas NA, Xia L, Wang X, Yang AD, Somcio RJ, Lin YG, Lim S, Fan F, Mangala LS, Arumugam T, Logsdon CD, Lopez-Berestein G, Sood AK, Ellis LM (2008). Therapeutic targeting of neuropilin-2 on colorectal carcinoma cells implanted in the murine liver. J Natl Cancer Inst.

